# Inhibition of 3‐Phosphoinositide‐Dependent Kinase 1 Improves Age‐Associated Epidermal Dysfunction

**DOI:** 10.1111/exd.70379

**Published:** 2026-10-30

**Authors:** Si‐Young Cho, Hyung‐Su Kim, Seung‐Hun Kim, Kyung‐Mi Joo, Sung Yeon Cho, Sang Wook Son, Heung‐Soo Baek, Hyoung‐June Kim, Jaeyoung Ko, A Young Kim

**Affiliations:** ^1^ Research and Innovation Center AMOREPACIFIC Yongin‐si Gyeonggi‐do Korea; ^2^ Department of Dermatology Korea University Ansan Hospital Ansan Korea

**Keywords:** autophagy, epidermal aging, epidermal homeostasis, kaempferol tetrasaccharide, PDK1

## Abstract

Epidermal aging is characterized by impaired homeostasis, including reduced cell proliferation, aberrant differentiation, deterioration of the dermal–epidermal junction (DEJ), and compromised barrier function. Accumulating evidence suggests that these alterations arise not solely from intrinsic stem cell loss but from disruption of regulatory signalling networks governing epidermal function. In this study, we investigate whether inhibition of 3‐phosphoinositide‐dependent kinase‐1 (PDK1), a central regulator of aging‐related AKT/mTOR and NF‐κB signalling, is associated with improved epidermal homeostasis through functional enhancement of autophagy. Using primary normal human epidermal keratinocytes (NHEKs) from young and old donors, 3‐dimensional organotypic epidermal skin equivalents (SE), and chronologically aged human skin, we found that aged epidermis exhibited markedly reduced autophagy activity accompanied by defective differentiation, impaired keratinocyte proliferation, and loss of key stem cell niche components at the DEJ. Pharmacological inhibition of PDK1 using selective PDK1 inhibitors (BX795, GSK2334470) or a plant‐derived inhibitor (kaempferol tetrasaccharide, KT1) formulated as *Camellia japonica* seed fraction (CF) suppressed AKT/mTOR and NF‐κB signalling in aged keratinocytes, which was accompanied by increased autophagy‐related markers and improved entry into differentiation. In addition, PDK1 inhibition increased basal keratinocyte proliferation, increased epidermal thickness, and improved DEJ components. In a proof‐of‐concept clinical study, topical application of CF, a botanical fraction containing KT1, was associated with improvements in epidermal autophagy, differentiation, DEJ integrity, and barrier function in aged human skin. Collectively, these findings identify a regulatory role of PDK1 within the epidermis and provide proof‐of‐concept evidence that pharmacological modulation of PDK1 activity exhibits potential for mitigating age‐associated epidermal dysfunction.

## 1 Introduction

Senescence of cutaneous tissue is a multifaceted biological phenomenon influenced by both intrinsic and extrinsic determinants, contributing to a progressive decline in physiological function and structural integrity [1]. Critical manifestations of this decline include diminished epidermal thickness, compromised hydration of the stratum corneum, attenuated wound repair, and suboptimal maintenance of the epidermal barrier, all of which reflect an underlying disruption of epidermal homeostasis due to aging [2, 3]. The epidermis serves as a dynamic, self‐renewing tissue that protects against microbial invasion and prevents undue water loss and is predominantly orchestrated by keratinocytes [4]. These cells exhibit proliferative capabilities within the basal layer, followed by the cessation of proliferation upon initial differentiation, leading to their ascension through various epidermal layers while undergoing both morphological transformation and differential gene expression relevant to terminal differentiation [5, 6]. The timeline and pace of epidermal turnover are closely regulated by homeostatic mechanisms, which become increasingly prolonged with age [7].

Fundamentally, the equilibrium of epidermal renewal is maintained by the regenerative capacity of basal keratinocytes and a meticulous balance between their proliferation and differentiation [8]. Age‐related deterioration of cutaneous homeostasis, manifested as reduced keratinocyte proliferation, aberrant differentiation, and altered epidermal structure, leads to increased irritant susceptibility, intensified xerosis, and pruritus development [9]. Emerging research indicates that such declines in homeostatic regulation are predominantly attributable not to inherent stem cell deficits within the basal layer but rather to a compromised microenvironmental niche characterized by dysfunction at the DEJ, derangements in the ECM, and aberrant metabolic signalling [8, 10]. Structural or functional impairments within the microenvironmental niche can adversely affect skin regeneration and repair capabilities. DEJ, featuring critical components such as Collagen type XVII (COL17) and laminins, secures epidermal cells in the dermis and is pivotal for stem cell‐mediated tissue renewal and homeostasis [8]. The ECM not only offers mechanical support but also regulates the behaviour of resident skin cells. Therefore, perturbations in the ECM architecture significantly affect the maintenance of the niche stability key for stem cell regulation [10]. Moreover, recent research has established a significant correlation between metabolic pathways, particularly autophagy, and the maintenance of epidermal homeostasis. Autophagy is increasingly recognized as a fundamental mechanism governing the homeostasis of epidermal lipids and plays a pivotal role in the facilitation of cell proliferation and the regulation of keratinocyte differentiation [11, 12, 13, 14]. Although multiple studies have shed light on microenvironmental determinants, such as Notch‐1 and mTOR signalling pathways and autophagy, an exhaustive molecular understanding of age‐related epidermal homeostasis impairment and its mitigation remains minimally elucidated.

PDK1, a serine/threonine protein kinase, is a critical component of the phosphatidylinositol 3‐kinase (PI3K) pathway and functions as a central regulator of cellular proliferation, apoptosis, immune responses, and cytoskeletal reorganization [15]. Consistent with this regulatory role, our previous studies showed that inhibition of PDK1 suppresses cellular senescence by suppressing NF‐κB and mTOR signalling pathways [16]. Furthermore, we demonstrated that CF, a plant‐derived PDK‐1 inhibitor containing KT1, effectively attenuates skin aging, particularly within the dermis. Upon topical administration, there was a notable improvement in collagen synthesis, a decrease in Matrix Metalloproteinase‐1 (MMP‐1) enzyme levels, and an increase in dermal density, collectively mitigating the symptoms of aging skin. Notably, CF and KT1 were also shown to promote keratinocyte proliferation in the basal layer of the epidermis [17]. However, the specific role of PDK1 signalling in chronological epidermal aging and its mechanistic link to autophagy remained unexplored. Based on these findings, we hypothesized that targeting the age‐related dysregulation of PDK1 signalling could improve disrupted epidermal homeostasis in aged skin.

To elucidate the role of PDK inhibition in maintaining epidermal homeostasis during skin aging, we first evaluated its inhibitory effects on keratinocyte proliferation and differentiation in aged 3D organotypic SE and chronologically aged skin. We revealed that inhibition of PDK1 was associated with improvement of age‐related defects in epidermal homeostasis, accompanied by modulation of AKT/mTOR and NF‐κB signalling and autophagy‐related activity. Subsequently, in a vehicle‐controlled clinical trial involving participants aged 60 and above, topical administration of CF was linked to improved skin barrier‐related parameters, including skin hydration and ceramide levels. Collectively, these findings suggest that PDK1 inhibition may have potential translational relevance for improving age‐associated epidermal dysfunction, while further studies are required to establish its clinical efficacy.

## 2 Materials and Methods

### 2.1 Materials

CF (RE.D Flavonoid; Amorepacific, Korea) and its active compounds, KT1, were prepared, purified, and analysed as described by Kim et al. [17]. Small‐molecule inhibitors of PDK‐1, including BX795 and GSK2334470, were purchased from Selleckchem (Houston, TX, USA). Rapamycin, Bafilomycin A1, and CaCl_2_ were obtained from Sigma‐Aldrich (St. Louis, MO, USA). The concentrations of each PDK1 inhibitor used in this study were carefully selected based on prior dose–response optimization experiments and previously published studies [17], including our own work, demonstrating effective modulation of PDK1 signalling without inducing cytotoxicity.

### 2.2 Cell Culture and Differentiation

NHEKs derived from different Caucasian donors (aged 14, 19, 21, 23, 58, 61, 62, and 68 years) were purchased from Lonza (Basel, Switzerland) and cultured in KGM‐Gold BulletKit Growth Media (Lonza) in a 5% CO_2_ incubator at 37°C. Differentiation was induced in confluent NHEKs cultures by supplementing the medium with 1.8 mM CaCl_2_ for 48 h. Inhibitor‐based mechanistic experiments were performed in representative donor‐derived NHEKs rather than across all donor lines.

### 2.3 Immunoblotting

Immunoblotting was performed as described previously [18]. Antibodies against LC3B, and Beclin‐1 were obtained from Novus Biologicals (Littleton, CO, USA); antibodies against phospho‐ULK1 (Ser555), ULK1, phospho‐AKT (Thr308), AKT, phospho‐4EBP1 (Ser65), 4E‐BP1, and β‐actin were purchased from Cell Signalling Technology (Beverly, MA, USA); an antibody against Filaggrin (FLG) from Invitrogen; antibodies against phospho‐NF‐κB (p65, Ser536), NF‐κB (p65), phosphor‐IκBα (Ser32/36), IκBα, and Involuclin (INV) were obtained from Abcam (Cambridge, MA, USA); antibody against p62 were purchased from MBL Life Science (Woburn, MA, USA), and antibodies against Keratin 10 (K10) and LOR were purchased from BioLegend (San Diego, CA, USA).

### 2.4 Autophagy Detection and Lysosomal Blockade Assay

Autophagic vacuoles and quantitative assessment of autophagic flux were evaluated using an Autophagy Detection Kit (Abcam) following the manufacturer's instructions. Briefly, NHEKs were washed with 1 × assay buffer and stained with microscopy dual detection reagent. After washing in 1 × assay buffer, the cells were fixed and stained with 4′,6‐diamidine‐2′‐phenylindole dihydrochloride (DAPI; Invitrogen). Fluorescence images were acquired using a confocal laser scanning microscope LSM800 and analysed using the Zen 2.5 imaging software (Zeiss, Oberkochen, Germany).

To assess lysosomal blockade‐dependent changes in autophagic processing, aged donor‐derived NHEKs were differentiated with 1.8 mM CaCl_2_ for 48 h in the presence or absence of PDK1 inhibitors (100 nM of BX795, 100 nM of GSK2334470, or 5 μg/mL of KT1) and Bafilomycin A1 (100 nM). Whole‐cell lysates were subjected to immunoblotting for p62, together with differentiation‐related and autophagy‐related markers.

### 2.5 Three‐Dimensional Organotypic Skin Equivalents Culture

Human epidermal models (Epiderm EPI‐201; MatTek Corporation, Ashland, MA, USA) obtained from donors of varying ages (21, 23, 58, and 62 years) were employed to evaluate the effects of PDK inhibition on 3D organotypic epidermal differentiation. Briefly, EPI‐201 tissues were cultured at the air‐liquid interface for 4 days in the presence or absence of BX‐795 (100 nM), GSK2334470 (100 nM), KT1 (5 μg/mL), and CF (10 μg/mL) to regenerate fully differentiated skin epidermis. Tissues were harvested on Day 6, fixed in 10% neutral buffered formalin for 4 h, and subsequently processed into paraffin blocks for further analyses.

### 2.6 Immunohistochemistry and Immunofluorescence on 3D Organotypic SE or Skin Sections

Immunohistochemical analysis of SE or skin sections was performed as previously described [17]. K10 antibody was purchased from BioLegend, and antibodies against FLG, INV, Integrin β1, and anti‐Ki‐67 were obtained from Abcam. For immunofluorescent staining, antigen retrieval was performed by heating the slides in Dako Target Retrieval Solution (Dako) for 20 min. Non‐specific staining was blocked by incubating the slides with PBS containing 10% donkey serum and 0.05% Triton X‐100 for 30 min. Tissue sections were incubated overnight at 4°C with primary antibodies against the COL17 (Abcam) and LC3B (Novus Biologicals) in Dako Fluorescence Mounting Medium (Dako). The tissue sections were then incubated with appropriate secondary antibodies conjugated with Alexa Fluor 488 (Invitrogen) for 2 h at room temperature. After washing in PBS, DAPI was applied for nuclear counterstaining. Coverslips were mounted on glass slides using a fluorescent mounting medium (Thermo Fisher Scientific, Waltham, MA, USA).

### 2.7 Efficacy of CF on the Buttock Skin of Older Women

A randomized, single‐blind, vehicle‐controlled study was performed to investigate the efficacy of CF on aged skin. Seven elderly (64.7 ± 2.4‐year‐old) and three young (32.3 ± 6.4‐year‐old) female volunteers with no current or history of skin disease were enrolled in the study. All participants provided informed consent. This clinical study was conducted in accordance with the Declaration of Helsinki, approved by the Institutional Review Board of Korea University Hospital (IRB: 2020AS0178), and registered at Clinical Research Information Service (CRIS; registration number: KCT0006565; First Posted: 12/11/2020) (https://cris.nih.go.kr/). The exclusion criteria were as follows: any medical condition that could influence the study results; known pregnancy or lactation; a history of oral immunosuppressive drug use or surgical procedures, including laser treatment within 3 months; and the use of topical medication within 4 weeks prior to study enrolment. All subjects received the CF (0.1%) and its vehicle (30% ethanol and 70% polyethylene glycol 400), which was applied to the buttock skin. Treatment (62.5 μL cm^−2^) was applied twice weekly for 3 weeks using a filter‐paper‐lined van der Bend Square Chamber (Van der Bend BV, Brielle, the Netherlands). The placement of the patch‐test chambers was randomized between the volunteers. Punch biopsy specimens (4 mm) were collected after 3 weeks from the test areas, 48 h following the final administration. The samples were fixed in 10% neutral buffered formalin and subsequently processed into paraffin blocks for further analysis.

### 2.8 A Vehicle‐Controlled Double‐Blinded Trial Comparing the Effects of 0.1% CF Cream and Vehicle in Subjects

To evaluate the effects of CF‐based cream on skin barrier function in aged human skin, a randomized, double‐blind, site‐randomized, intra‐individual, vehicle‐controlled study was conducted with 24 healthy female participants aged 60–69 years (average = 63.2 ± 2.40). Among the 24 participants who initiated the study, 20 completed the study. Four subjects withdrew owing to non‐compliance. Throughout the investigation, no instances of adverse dermatological effects were reported among any of the participants. This clinical study was conducted at the P&K Skin Research Center (Seoul, Korea) in compliance with the principles outlined in the Declaration of Helsinki. Written informed consent was obtained from all participants after a comprehensive explanation of the study. The study was approved by the Institutional Review Board of the P&K Skin Research Center (P2103‐1868) and registered with the CRIS (registration number: KCT0006228; First Posted: 02/04/2021). Following randomization, participants were assigned to receive either topical 0.1% CF cream or a vehicle (base formulation without CF; Table S1) applied to designated square areas (dimensions: 3 × 5 cm) on both forearms twice daily for 4 weeks. The test areas were randomly allocated to the forearms of each participant. Improvements in skin moisture, water loss, and skin transparency were measured at baseline and at Weeks 2 and 4 with a Corneometer CM 825 (Courage & Khazaka GmbH, Germany), a vapometer (SWL4001, Delfin Technologies Ltd., Kuopio, Finland), and translucency meter (TLS850, Diastron Ltd., Hampshire, UK) on the right forearm. For safety evaluation, tolerability was measured using a subject self‐assessment questionnaire with a four‐point scale at baseline and 4 weeks in the entire population.

### 2.9 Lipid Extraction and Analysis of the Ceramide Content Using the Tape Strips

Each treatment area was tape stripped once using 10 consecutive tapes (3.8 cm^2^, D‐Squame; Monaderm, Monaco, France) at the 4th week. Tapes were applied at the center of one of the square angles of all test areas to avoid interference in the measurement of skin hydration. After application of a pressure applicator (225 g/cm^2^) (D‐squame; Monaderm, Monaco, France) for approximately 10 s, the tape was gently removed and stored at −20°C until further analysis. Half of the adhesive tape was used for lipid extraction. This segment was submerged in a mixture containing 3 mL of ethyl acetate and methanol in a volumetric ratio of 1:2, followed by sonication for a duration of 10 min to facilitate extraction. Upon completion, the organic phase was isolated and subjected to a drying process using a SpeedVac vacuum concentrator (model EZ‐2 Plus; Genevac, Switzerland), and the temperature parameters were set to 40°C. The resultant dry residues were then reconstituted in a 200 μL solution of methanol and chloroform mixed in a 2:1 volumetric ratio, and thoroughly mixed by vortexing for 5 min. Ensuing this step, the mixture underwent centrifugation at 17 800 × *g* at 4°C for 5 min, a prerequisite for subsequent analysis by Ultra‐Performance Liquid Chromatography‐Tandem Massrometry (UPLC‐MS/MS) utilizing an ACQUITY UPLC system coupled to a Xevo TQ‐S mass spectrometer (Waters Corp, Milford, MA, USA). This analytical process enabled the quantification of four distinct ceramide subclasses: non‐hydroxy fatty acid (FA) conjugated to phytosphingosine (NP), non‐hydroxy FA conjugated to sphingosine (NS), non‐hydroxy FA conjugated to dihydrosphingosine (NDS), and α‐hydroxy FA conjugated to phytosphingosine (AP), which were sourced from Avanti Polar Lipids (Alabaster, AL, USA) and measured in accordance with an established UPLC‐MS/MS protocol.

### 2.10 Statistical Analysis

Statistical analyses were performed using biological replicates as the unit of analysis, as specified for each experiment. For cell‐based experiments, NHEKs from representative young and aged donors were analysed with three independent experimental repeats (*n* = 3 experimental repeats per donor; *n* does not represent independent donor‐level biological replicates). For 3D skin equivalent experiments, three independent skin equivalents were analysed per group (*n* = 3 per group), with image‐level values averaged within each tissue prior to analysis. For the patch test study, 10 human subjects were analysed (young, *n* = 3; aged, *n* = 7), with each subject treated as an independent biological replicate. For the randomized controlled trial, analyses were performed at the subject level in 20 participants (*n* = 20).

Data were analysed using the unpaired two‐sided Student's *t*‐test or one‐way analysis of variance (ANOVA), followed by Dunnett's multiple comparisons test to determine statistical significance, after confirming that the data met the appropriate assumptions (normality, independent sampling, and homogeneity of variance). For the randomized controlled trial, paired statistical analyses were used to account for the intra‐individual study design. Repeated measurements of skin hydration, TEWL, and skin transparency were analysed using a two‐way repeated measures ANOVA with treatment and time as within‐subject factors. The main effects of treatment and time and the treatment × time interaction were evaluated, with Bonferroni correction for multiple comparisons. Changes in stratum corneum ceramide levels from baseline to Week 4 were compared between the paired CF‐treated and vehicle‐treated sites using a paired, two‐tailed Student's *t*‐test. For other nonparametric analyses, the Friedman test and Mann–Whitney *U* test were used to compare within‐group and between‐group data, respectively. For statistical analysis of the ceramide data, an unpaired *t*‐test was used to compare the groups 4 weeks after application. Data analyses were performed using SPSS version 20 (SPSS Inc., Chicago, IL, USA). Statistical significance was assumed at *p < 0.05, **p < 0.01, and ***p < 0.001. All data are presented as the mean ± standard deviation.

## 3 Results

### 3.1 PDK1 Inhibition Ameliorates Epidermal Proliferation and Stem Cell Niche Integrity in Aged Skin

Based on our previous observation that PDK1 inhibition increased epidermal thickness and Ki‐67 positive basal keratinocytes in aged human skin [17], we examined whether PDK1 directly regulates epidermal proliferation and function during aging. To verify the role of PDK1 on epidermal aging, we established epidermal SE and analysed the aging‐related changes of DEJ components and their effects on epidermal homeostasis, including self‐renewal and differentiation of basal cells. Consistent with previous results, SE derived from old donors exhibited a thinner epidermis with fewer basal keratinocytes expressing Ki‐67 (Figure 1, Figure S1). Inhibition of PDK1 activity using BX795, GSK2334470, selective PDK1 inhibitors, or KT1, a PDK1 inhibitor derived from a phytochemical origin, increased the reduced Ki‐67‐positive proliferating basal cells and thin epidermal layer in SE derived from aged donors (Figure 1).

**FIGURE 1:**
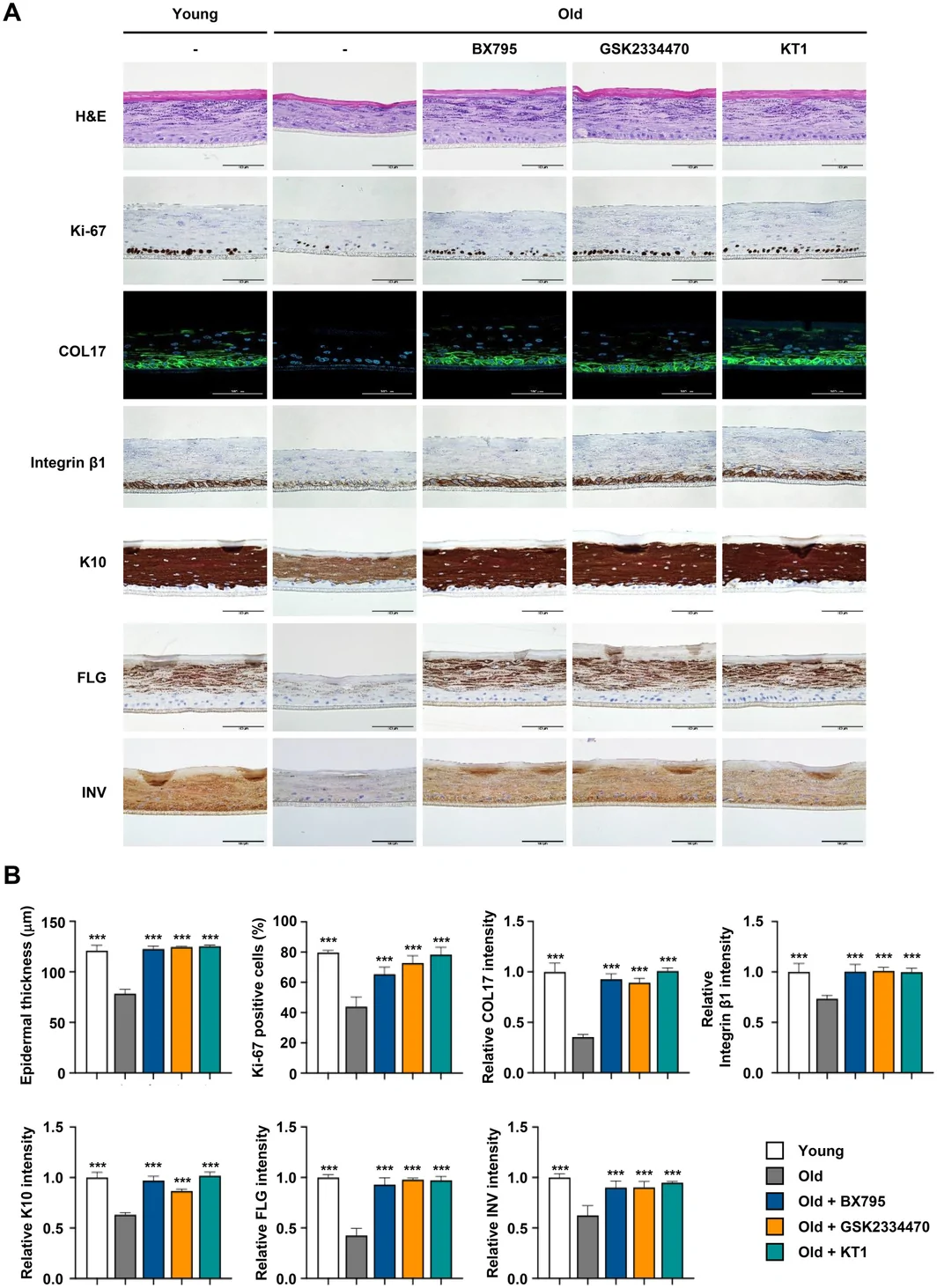
Inhibition of PDK1 ameliorates age‐related changes of epidermal homeostasis. (A) Age‐related changes of DEJ components and their effects on epidermal homeostasis in 3D SE derived from young and old donors. Skin equivalent sections were stained with haematoxylin and eosin (H&E). Proteins of Ki‐67, COL17, Integrin β1, K10, FLG, and INV were detected using immunohistochemistry and immunofluorescence staining. Scale bars, 100 μm. (B) Quantification of the epidermal thickness, Ki‐67‐positive cells, and the levels of COL17, Integrin β1, K10, FLG, and INV. Three representative images were analysed per skin equivalent, and image‐level values were averaged to yield one value per tissue for statistical analysis (*n* = 3 independent skin equivalents per group). ***p < 0.001 compared to the vehicle‐treated old group. Young, SE derived from 21 year donor; Old, SE derived from 58 year donor.

To further evaluate the functions of PDK1 in the epidermis, we checked several key markers that regulate epidermal homeostasis, including self‐renewal and differentiation of basal keratinocytes. As observed in the aged human epidermis [19, 20, 21], the expression levels of Collagen XVII (COL17) and Integrin β1, key components of the epidermal stem cell niche that support epidermal progenitor cell expansion, were significantly decreased in SE derived from old donors compared to those derived from young donors (Figure 1, Figure S1). Consistently, keratinocyte differentiation markers such as K10, FLG, and IVL were decreased in SE derived from old donors compared to those from young donors (Figure 1, Figure S1). Surprisingly, the treatment of PDK1 inhibitors increased the levels of COL17, Integrin β1, K10, FLG, and IVL in SE derived from an old donor (Figure 1). These results indicate that inhibition of PDK1 is associated with partial improvement of age‐associated defects in epidermal homeostasis, as reflected by increased basal keratinocyte proliferation, improved epidermal differentiation, and preservation of stem cell niche components independently of dermal effects.

### 3.2 PDK1 Inhibition Suppresses AKT/mTOR and NF‐κB Signalling in Aged Keratinocyte

Our previous findings report that inhibition of PDK1 in senescent human dermal fibroblasts reverts senescent phenotypes through suppressing both mTOR signalling and NF‐κB signalling [16]. To examine whether PDK1 is involved in age‐induced disruption of epidermal homeostasis by modulating mTOR and NF‐κB signalling pathways, we checked key proteins in these signalling pathways. Upon CaCl_2_‐induced differentiation, NHEKs derived from old donors exhibited significantly lower expression of keratinocyte differentiation markers, such as K10 and INV, than those derived from young donors (Figures 2A,B, Figure S2). However, inhibition of PDK1 with 3 different PDK1 inhibitors increased the levels of these keratinocyte markers (Figure 2A,B). In line with previous findings [16], PDK1 inhibition also decreased upregulated phosphorylation levels of AKT and eukaryotic translation initiation factor 4E‐binding protein 1 (4EBP1) in aged NHEKs (Figure 2A,B). Moreover, enhanced NF‐κB activity in aged NHEKs was reduced by PDK1 inhibitor treatment (Figures 2C,D). Collectively, these findings suggest that PDK1 inhibition alleviates aging‐induced epidermal dysfunction by suppressing AKT/mTOR and NF‐κB signalling, thereby restoring aging‐induced dysregulation of epidermal homeostasis.

**FIGURE 2:**
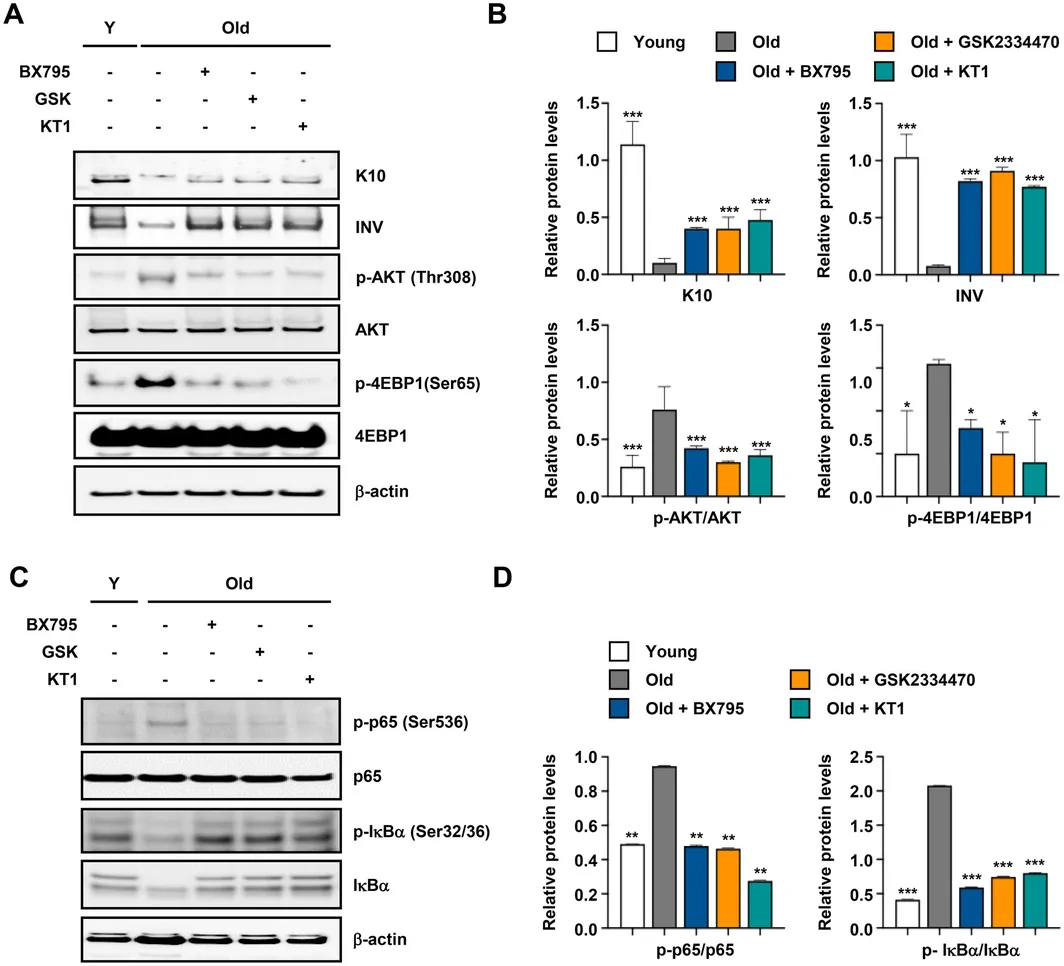
PDK1 inhibition attenuates AKT/mTOR and NF‐κB activity in old donor‐derived NHEKs. (A) Western blotting of differentiation‐related proteins and AKT/mTOR signalling in aged NHEKs treated for 48 h with two selective PDK1 inhibitors (100 nM of BX795, 100 nM of GSK2334470) or the natural PDK1 inhibitor, KT1 (5 μg/mL), during CaCl_2_‐induced differentiation. (B) Quantification of K10, INV and AKT/4EBP1 phosphorylation levels. (C, D) Western blotting of p65 and IκBα phosphorylation in NHEKs after treatment of PDK1 inhibitors for 6 h. (D) Quantification of p65 and IκBα phosphorylation levels. Data represent the mean of three independent experimental repeats (*n* = 3) performed using NHEKs from a representative young donor (21 year) and an aged (58 year) donor; *n* does not represent independent donors. Western blots shown are representative of these independent experiments. *p < 0.05; **p < 0.01; ***p < 0.001 compared to the vehicle‐treated old NHEKs. Y, NHEKs from 21 year donor; Old, NHEKs from 58 year donor.

### 3.3 Autophagic Activity Is Diminished in Aged Keratinocytes and Is Associated With Impaired Epidermal Differentiation

Given the essential role of autophagy in epidermal homeostasis, especially keratinocyte differentiation [14], and autophagy function declines with age in several tissues [22], we next assessed whether autophagic activity is altered in keratinocytes derived from aged donors during differentiation. Young donor‐derived NHEKs exhibited increased levels of Unc‐51‐like autophagy activating kinase 1 (ULK1) phosphorylation, Beclin1, and Microtubule‐associated protein 1 light chain 3 beta (LC3B) after the addition of calcium chloride, compared to old donor‐derived NHEKs (Figure S3). Furthermore, CaCl_2_‐mediated keratinocyte differentiation induced autophagosome formation in young donor‐derived NHEKs but not in old donor‐derived NHEKs (Figure S3). To further assess the relationship between differentiation and autophagy according to age, the effects of autophagy inducers or inhibitors on keratinocyte differentiation in young and old donor‐derived NHEKs were examined. Treatment of autophagy inhibitor Bafilomycin A1 into young donor‐derived NHEKs inhibited the expression of differentiation markers, such as K10 and INV, during CaCl_2_‐induced differentiation (Figure S4). In contrast, rapamycin, an autophagy inducer, rescued differentiation defects in old donor‐derived NHEKs (Figure S4), indicating that the loss of autophagic activity in old donor‐derived NHEKs is associated with impaired keratinocyte differentiation.

### 3.4 PDK1 Inhibition Enhances Autophagy and Promotes Differentiation in Aged Keratinocytes

The finding that the inhibition of PDK1 activity blocks AKT/mTOR activity, thereby enhancing autophagy [23], led us to investigate the effects of PDK1 inhibition on epidermal homeostasis through the modulation of autophagy in aged keratinocytes. Strikingly, treatment with PDK1 inhibitors increased the phosphorylation levels of ULK1 and the protein levels of Beclin1 and LC3B in old donor‐derived NHEKs (Figure 3A,B). Reduced autophagosome formation during differentiation was also alleviated by PDK1 inhibition in aged NHEKs (Figures 3C,D). In addition, down‐regulated LC3B level in SE derived from aged donors compared with SE derived from young donors was improved by PDK1 inhibition (Figure 3E,F). To further assess whether these effects were autophagy‐dependent, lysosomal inhibition with Bafilomycin A1 was applied in aged keratinocytes. Bafilomycin A1 attenuated the differentiation‐promoting effects associated with PDK1 inhibition (Figure S5). Consistent with these observations, the age‐related accumulation of p62 was reduced by PDK1 inhibitors, and this reduction was blocked by Bafilomycin A1, supporting functional enhancement of autophagic processing (Figure S5). These findings suggest that intact lysosomal/autophagic processing may contribute to the differentiation‐associated effects of PDK1 inhibition. Collectively, our results support an association between PDK1 inhibition and enhanced autophagy‐related activity in aged keratinocytes, potentially facilitating their progression toward differentiation.

**FIGURE 3:**
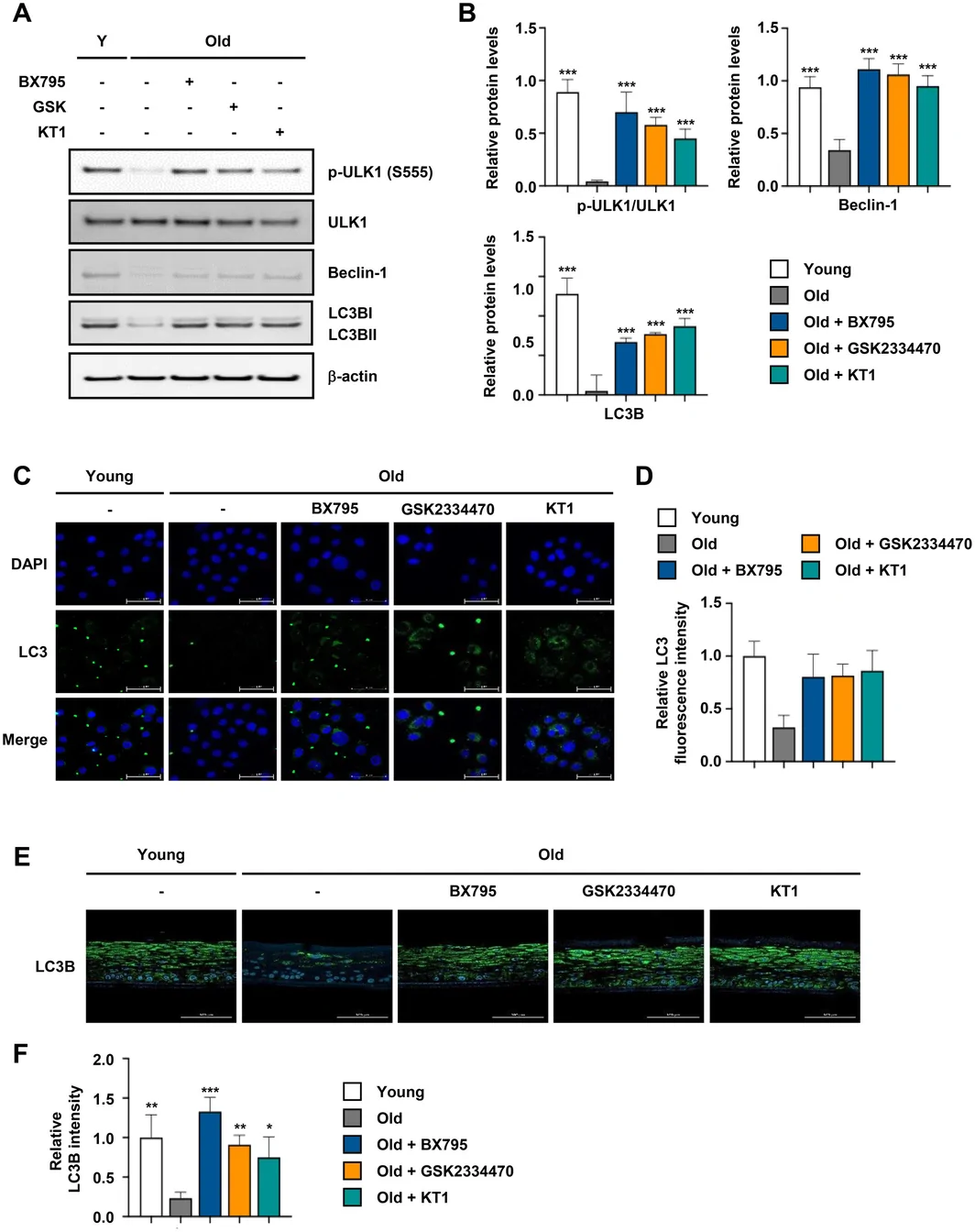
PDK1 inhibition is associated with enhanced autophagy‐related activity and improved age‐impaired keratinocyte differentiation. (A) Western blotting of autophagy‐related proteins in aged NHEK treated for 48 h with PDK1 inhibitors, BX795 (100 nM), GSK2334470 (100 nM), or KT1 (5 μg/mL), during CaCl_2_‐induced differentiation. (B) Quantification of ULK1 phosphorylation, Beclin1, and LC3B levels. (C) Immunofluorescence staining of LC3‐positive autophagosomes in aged NHEKs treated with BX795 (100 nM), GSK2334470 (100 nM), or KT1 (5 μg/mL). Scale bars, 50 μm. (D) Quantification of mean LC3 fluorescence intensity. (E) Histological analysis of epidermal skin equivalent model. LC3B was detected using immunofluorescence staining. Scale bars, 100 μm. (F) Quantification of LC3B using ImageJ software. (A–D) Data represent the mean of three independent experimental repeats (*n* = 3) performed using NHEKs from a representative young donor (21 year) and an aged (58 year) donor; *n* does not represent independent donors. (E and F) Three representative images were analysed per skin equivalent, and image‐level values were averaged to yield one value per tissue for statistical analysis (*n* = 3 independent skin equivalents per group). *p < 0.05; **p < 0.01; ***p < 0.001 compared to the vehicle‐treated old NHEKs or vehicle‐treated old SE. Y and Young, NHEKs from 21 year donor or SE derived from 21 year donor; Old, NHEKs from 58 year donor or SE derived from 58 year donor.

### 3.5 Topical CF Treatment Improves Altered Epidermal Homeostasis of Chronologically Aged Skin

To assess the clinical implications of PDK1 inhibition in chronologically aged human skin, we tested CF (containing 67% KT1, Figure S6), a natural PDK1 inhibitor, as an alternative to synthetic inhibitors. Consistent with previous results, CF treatment was associated with improvement of age‐related changes in epidermal differentiation and autophagy‐related markers in NHEKs and 3D organotypic SE (Figures S7 and S8). In a 3‐week randomized, single‐blind, vehicle‐controlled pilot study (7 elderly and 3 young adults), aged skin showed prominent alterations at the DEJ and in the basal cell layer (Figure 4A,B). Topical treatment of CF ameliorated these aging features, increasing epidermal thickness, the number of Ki‐67 positive cells, and the levels of COL17 and Integrin β1 (Figures 4A,B). Furthermore, topical application of CF led to increased LC3B and higher expression of skin barrier‐related proteins such as K10 and FLG (Figure 4A,B). These results suggest that CF ameliorates epidermal atrophy of aged skin, including the upregulation of stem cell niche components, autophagy, and skin barrier‐associated proteins.

**FIGURE 4:**
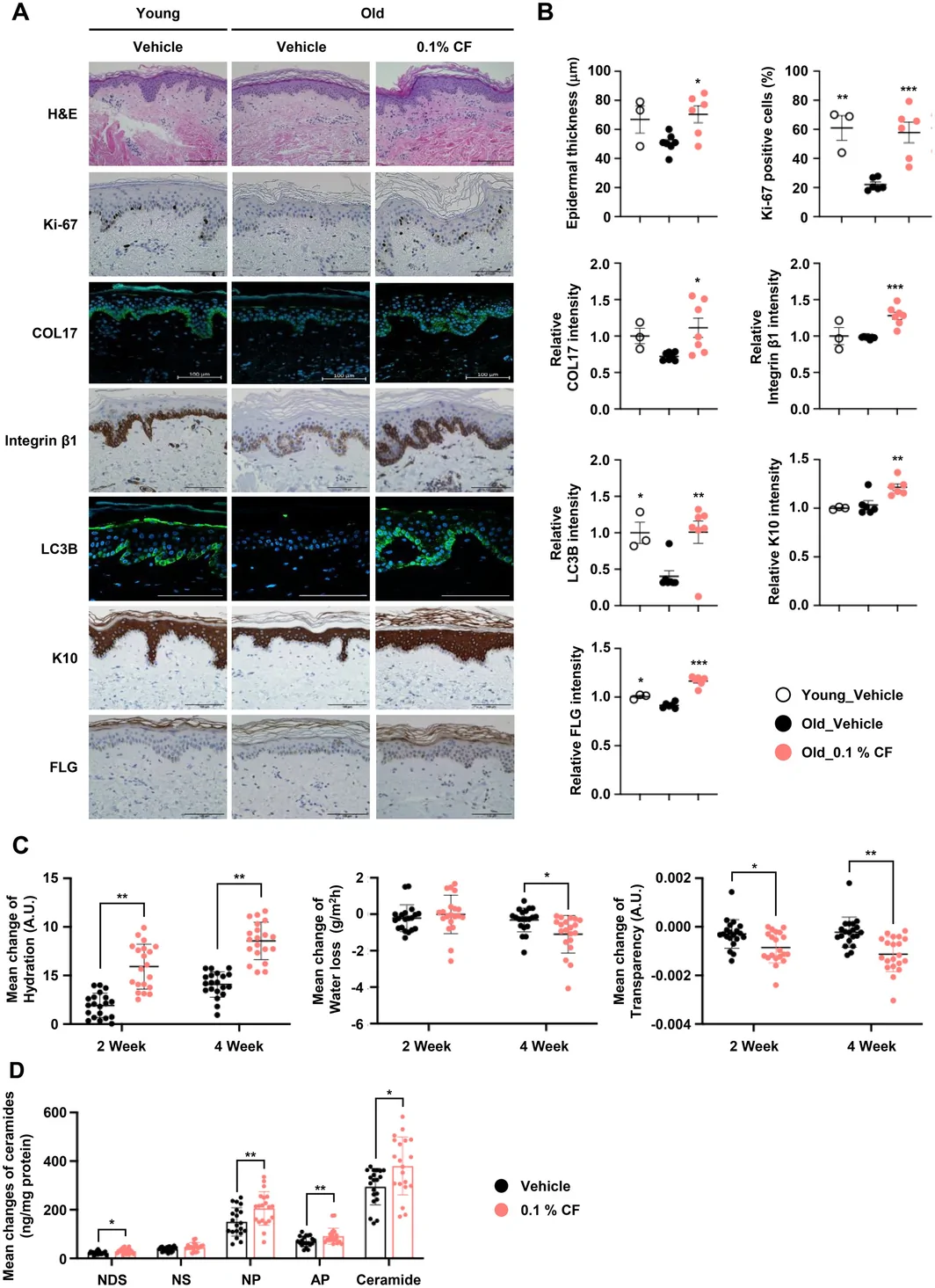
Clinical effects of PDK1 inhibition on age‐related alteration of epidermal homeostasis and skin barrier impairments. (A) Representative histological and immunofluorescent images from a 3‐week randomized, single‐blind, vehicle‐controlled study in 10 participants comparing topical 0.1% CF treatment between young (*n* = 3) and old (*n* = 7) donors. Scale bars, 100 μm. (B) Quantification of epidermal thickness, Ki‐67‐positive cell ratios, and the levels of COL17, Integrin β1, LC3B, K10, and FLG. A total of 10 human subjects were analysed (young: *N* = 3; aged: *N* = 7). Quantification was performed across all participants, with each subject treated as an independent biological replicate. (C and D) Clinical effects of CF on skin barrier impairments in older women's forearms. (C) Changes in skin hydration, water loss, and transparency from baseline after 2 and 4 weeks of vehicle or CF‐treated group were evaluated. (D) Changes in ceramide contents in the stratum corneum from baseline after 4 weeks of vehicle or CF‐treated group. A total of 20 participants completed the randomized controlled trial, and all statistical analyses were performed at the subject level (*n* = 20).*p < 0.05; **p < 0.01; ***p < 0.001 compared to the vehicle‐treated old group.

### 3.6 Topical Treatment of CF Improves the Skin Barrier Function in Older Women

Based on the data from the 3D organotypic SE and 3‐week occlusive application of CF on aged skin, we hypothesized that CF would improve age‐associated epidermal barrier dysfunction, thereby improving skin hydration in the elderly. To test this possibility, a 4‐week, randomized, double‐blind, vehicle‐controlled study was conducted on 20 elderly adults (Figure S9). Two‐way repeated‐measures ANOVA showed significant treatment × time interactions for skin hydration, TEWL, and skin transparency (Figure 4C and Table S2), indicating that changes over time differed significantly between the CF‐ and vehicle‐treated sites. The CF‐treated sites showed increases in skin hydration and transparency and a reduction of TEWL over the treatment period. To assess alterations in ceramide concentrations within the stratum corneum following topical administration of CF, we quantified major ceramide species, including NP, NS, NDS, and AP. Paired analysis showed increases from baseline in NP, NDS, AP, and total ceramide levels after 4 weeks (Figure 4D). These findings suggest that CF treatment is associated with improvements in epidermal barrier‐related parameters and hydration in aged skin, supporting its potential translational relevance as exploratory proof‐of‐concept evidence.

## 4 Discussion

It is widely acknowledged that the complex equilibrium of epidermal homeostasis constitutes a fundamental axis for preserving skin integrity and function [24, 25]. This equilibrium can be disturbed by inflammatory cutaneous pathologies or the chronological progression of aging, culminating in compromised barrier function and suboptimal wound repair [26, 27]. Building upon our previous study demonstrating that PDK1 inhibition mitigates cellular senescence in dermal fibroblasts and ameliorates the appearance of facial wrinkles [17], this study represents a substantial incremental advance by specifically delineating the mechanisms of age‐associated epidermal dysfunction. Although our prior work established the anti‐senescence effects of PDK1 inhibition in the dermis and whole‐skin atrophy, the present study supports the involvement of PDK1‐related signalling in epidermal homeostasis during chronological aging. Although PDK1 has been reported to support epidermal stratification and Notch‐dependent differentiation during development by organizing asymmetric cell division, our findings suggest that in aged keratinocytes [28], dysregulated PDK1 activity may instead contribute to impaired autophagic flux and defective differentiation. Specifically, our data are consistent with the possibility that attenuation of PDK1/AKT/mTOR signalling supports functional enhancement of autophagic processing, thereby contributing to the improvement of terminal differentiation and barrier homeostasis. Because keratinocytes exhibit fast turnover, with aging predominantly affecting stem cell populations and terminal differentiation, a process heavily reliant on autophagy for eliminating organelles to maintain barrier integrity [29, 30, 31, 32, 33]. Thus, the present findings provide supportive evidence linking PDK1‐related signalling, autophagy‐associated regulation, and epidermal homeostasis in the context of skin aging, expanding upon the general AKT/mTOR/NF‐κB signalling paradigm explored in our prior work.

Although the PDK1‐AKT pathway typically drives cell proliferation, the increase in Ki‐67‐positive basal keratinocytes following PDK1 inhibition should be interpreted in the context of the aged epidermis. Sustained activation of the PI3K/AKT/mTOR axis can induce stress‐mediated cell‐cycle arrest, a phenomenon referred to as AKT‐induced senescence [34]. Therefore, downregulating PDK1 activity likely mitigates this dysregulated signalling. By alleviating senescence‐driven mitotic arrest, PDK1 inhibition may allow dormant basal keratinocytes to regain their proliferative competence. This suggests a potential mechanism for the elevated Ki‐67 levels and re‐establishing a normalized proliferative pool. Consequently, this enhanced progenitor capacity may support a more balanced renewal‐to‐differentiation trajectory, improving the structural integrity of the aged epidermis rather than inducing hyperproliferation.

We also explored changes within the structural environment of the epidermis, highlighting the reversibility of age‐induced alterations in the stem cell niche, specifically at the DEJ. DEJ is central to epidermal homeostasis, and its decline owing to intrinsic aging and UV‐induced damage are well‐established predictors of skin aging [35, 36]. With aging, the degradation of collagen and laminin, accompanied by reduced COL17A1 expression and DEJ flattening, results in the disruption of DEJ integrity, consequently affecting the regenerative potential of the basal keratinocytes and disrupting epidermal homeostasis, which collectively compromise skin resilience and impair wound healing [19, 26, 37, 38]. Treatment with PDK1 inhibitors was associated with preservation of critical components such as COL17 and Integrin β1, which are important for progenitor cell dynamics. This preservation may be related, at least in part, to attenuation of NF‐κB signalling, which mitigates degradation and maintains the structural integrity of the epidermal–dermal interface.

The translational relevance of our findings is underscored by observations following the topical application of CF, which contains KT1 and showed PDK1 inhibitory activity in our in vitro studies. In an aged skin model, CF treatment was associated with attenuation of epidermal thinning and increases in keratinocyte proliferation and differentiation markers, consistent with functional improvement in age‐related structural skin parameters. Consistent with these preclinical findings, small exploratory clinical studies in elderly subjects showed that CF intervention improved epidermal barrier‐related parameters, including hydration, skin transparency, and ceramide levels. These findings are also in line with recent studies showing that age‐related alterations in epidermal lipid composition are associated with impaired barrier function [39] and that specific ceramide subclasses are closely associated with skin barrier quality and hydration status [40]. These functional improvements may be associated with multiple biological effects of CF, including possible modulation of PDK1‐related signalling and autophagy‐associated processes. However, direct PDK1‐target engagement was not assessed in vivo. In support of this possibility, a previous clinical study showed that enhanced autophagy correlated with reduced trans‐epidermal water loss and increased skin hydration [41]. Collectively, these findings indicate that age‐associated epidermal barrier impairment may be amenable to strategies aimed at improving epidermal homeostasis and support further investigation of CF and PDK1‐related pathways in this context. However, the present clinical observations should be interpreted as exploratory proof‐of‐concept evidence rather than definitive evidence of clinical efficacy.

To the best of our knowledge, this study is the first to report on the inhibition of PDK1 as a strategy to mitigate epidermal aging. Despite its notable findings, this study has several limitations. First, the clinical component of this study should be interpreted cautiously. The clinical efficacy of selective small‐molecule PDK1 inhibitors like BX795 and GSK2334470 could not be thoroughly evaluated in humans owing to safety considerations. Instead, the translational studies used CF, a natural fraction containing KT1 that showed PDK1 inhibitory activity in vitro, and CF was confirmed to penetrate up to the stratum corneum layer. Although these findings support the feasibility of topical application of CF, direct engagement of PDK1 by CF was not demonstrated in vivo, and the clinical studies were exploratory in nature, with modest sample sizes, short treatment durations, and endpoints limited to proxy markers of epidermal barrier function. Furthermore, all study participants were female with Asian skin. Therefore, the clinical observations should be interpreted as exploratory proof‐of‐concept evidence supporting the potential translational relevance of CF treatment, rather than direct evidence of PDK1 inhibition in vivo or clinical efficacy or broad demographic applicability. In addition, the translational and exploratory clinical observations relied on CF, a proprietary natural extract evaluated within a single industrial research center, and the study did not include a head‐to‐head comparison with established dermatological standards, such as topical retinoids. Future independent replication, along with comparative efficacy studies of CF and appropriately validated PDK1‐targeted approaches against standard‐of‐care anti‐aging therapies, will be essential to fully contextualize their clinical utility and position within the current dermatological paradigm. Considering the intricacies of aging, further research should focus on larger, longer‐term studies incorporating clinically meaningful outcomes such as wrinkle severity and skin elasticity. It will also be vital to investigate the efficacy of PDK1 inhibitors across broader demographics, including male participants and other ethnic skin types, their impact on individuals with skin diseases, and their potential synergistic application with established dermatological therapies. Second, the inhibitor‐based experimental design has inherent limitations. Although we employed multiple structurally and mechanistically distinct inhibitors that are known to suppress PDK1 activity, we cannot entirely rule out the possibility that off‐target effects of the chemical inhibitors contributed to the observed outcomes. BX795 is known to inhibit kinases other than PDK1, including TBK1 and IKKε [42], and the broader kinase selectivity profile of KT1 has not been fully established. Therefore, BX795‐ or KT1‐related effects cannot be attributed solely to PDK1 when considered in isolation. In contrast, GSK2334470 is a well‐characterized PDK1 inhibitor with relatively high selectivity for PDK1. Importantly, the convergence of phenotypes across these three distinct inhibitors, including suppression of AKT/mTOR signalling, increased autophagy‐related markers, improved differentiation markers, and partial normalization of DEJ‐associated markers, supports the involvement of a PDK1‐related signalling axis. These findings should therefore be interpreted as supportive rather than definitive evidence of the involvement of the PDK1 signalling axis. Third, a notable limitation of our study is that while baseline age‐associated differences were evaluated across the entire cohort of donor‐derived NHEKs, the subsequent inhibitor‐based mechanistic and lysosomal flux experiments were conducted using representative donor samples. Consequently, although these donor models support the proposed pathways, they do not fully account for potential donor‐to‐donor biological variability. Therefore, studies incorporating larger donor panels will be necessary to systematically assess interindividual variability and validate these findings across a broader population. Fourth, the mechanistic conclusions remain limited by the absence of direct genetic validation. Although the Bafilomycin A1/p62 experiments suggest that intact lysosomal/autophagic processing contributes to the differentiation‐associated effects of PDK1 inhibition, the current evidence remains largely pharmacological and does not definitively establish a causal genetic pathway. Thus, genetic perturbation of PDK1 and essential autophagy‐related genes, such as ATG5 or ATG7, will be required to directly determine whether autophagic processing mediates these effects on epidermal differentiation and homeostasis. Fifth, the role of reduced inflammatory signalling in the observed improvement in DEJ‐associated proteins and epidermal homeostasis remains unclear. Although attenuation of NF‐κB signalling was observed, representative inflammatory mediators and senescence‐associated secretory phenotype (SASP)‐associated factors were not directly quantified in the present study. Therefore, whether reduced inflammatory mediator production underlies the preservation of DEJ‐associated proteins or improvement of epidermal function has not been established. Future studies measuring NF‐κB target genes and SASP components, including IL‐6, IL‐8, and TNF, will be required to determine the functional relationship between inflammatory attenuation and the epidermal effects of PDK1 inhibition in aged skin.

In summary, multiple factors compromise the aging epidermis, leading to the deterioration of physiological functions and structural integrity. Our data suggest that PDK1 inhibition is associated with improvement of several cellular and tissue‐level features of aged skin, including keratinocyte differentiation, stem cell niche markers, autophagy‐related activity, and epidermal barrier‐linked parameters. These findings support PDK1‐related signalling as a potential regulatory axis in aged epidermal homeostasis and provide proof‐of‐concept evidence for further investigation of PDK1‐targeted approaches. Considering the complexity of aging, further research should focus on the long‐term safety, clinical effectiveness, mechanistic validation using genetic approaches, broader demographic applicability, and potential combination with established dermatological therapies.

## Author Contributions

S.‐Y.C. performed the biochemical assays and immunostaining; J.K. isolated and identified C. flavonoids and flavonol compounds; K.‐M.J. performed the lipid analysis; H.‐S.K., S.‐H.K., S.W.S., and S.‐Y.C. conducted the clinical research; S.‐Y.C. and H.‐S.K. designed the study; S.‐Y.C., H.‐S.K., and A.Y.K. wrote the manuscript; H.‐J.K. and H.‐S.B. conceived the study; J.K. and A.Y.K. conceived and designed the research, analysed and interpreted the data, and oversaw the project. All authors approved the submitted manuscript version.

## Conflicts of Interest

The authors are employees of Amorepacific Corporation. CF/RE.D Flavonoid is a proprietary preparation developed by Amorepacific Corporation, which has a commercial interest in the preparation evaluated in this study.

## Supporting information


**Figure S1:** Age‐related changes of DEJ components and their effects on epidermal homeostasis in 3D SE derived from young and old donors. (A) SE sections were stained with H&E. The indicated proteins were detected by immunohistochemistry or immunofluorescence staining. Results are from one representative experiment out of three. Scale bars, 100 μm. (B) Quantification of epidermal thickness, Ki‐67‐positive cells, and the levels of COL17, Integrin β1, K10, FLG, INV, and LC3B using ImageJ software. Three representative images were analysed per skin equivalent, and image‐level values were averaged to yield one value per tissue for statistical analysis (*n* = 3 independent skin equivalents per group). All results are expressed as mean ± SD. *p < 0.05; **p < 0.01; ***p < 0.001 compared to the young donors. SD, standard deviation.
**Figure S2:** Age‐associated changes in keratinocyte differentiation during CaCl_2_‐induced differentiation of NHEKs. (A) Immunoblotting of differentiation markers in NHEKs isolated from young and aged donors after CaCl_2_ stimulation. The β‐actin panel is identical to that shown in Figure S3A, as the proteins presented in both figures were analysed using the same experimental samples and shared loading control. (B) Quantification of K10 and INV levels. Data are presented as a donor‐level scatter plot, where each point represents an individual donor (*n* = 4 per age group). All results are expressed as mean ± SD. ** *p* < 0.001 compared to the young donors. SD, standard deviation.
**Figure S3:** Age‐associated changes in autophagy during CaCl_2_‐induced differentiation of NHEKs. (A) Immunoblotting of autophagy markers in NHEKs isolated from young and aged donors after CaCl_2_ stimulation. The β‐actin panel is identical to that shown in Figure S2A, as the proteins presented in both figures were analysed using the same experimental samples and shared loading control. (B) Quantification of ULK1 phosphorylation, Beclin1, and LC3B levels. Data are presented as a donor‐level scatter plot, where each point represents an individual donor (*n* = 4 per age group). (C) Autophagosome formation visualized by immunofluorescence staining of LC3‐positive structures in NHEK from young versus aged donors. Scale bars, 50 μm. (D) Quantitative fluorescence intensity of the autophagosome. Data represent the mean of three independent experimental repeats (*n* = 3). All results are expressed as mean ± SD. ** *p* < 0.01; *** *p* < 0.001 compared to the young donors. SD, standard deviation.
**Figure S4:** Autophagy inhibition impairs, whereas autophagy induction restores, keratinocyte differentiation during CaCl_2_‐induced differentiation conditions. (A) Western blotting of differentiation and autophagy markers in NHEKs. Bafilomycin A1 (Bafilomycin, 100 nM) was co‐treated to inhibit autophagy in NHEKs from young donor. Rapamycin (250 nM) was used to induce autophagy in NHEKs from old donors. (B) Quantification of K10, INV, Beclin1, and LC3B levels. (C) Autophagosome formation visualized by immunofluorescence staining in NHEK from young and aged donors treated with bafilomycin or rapamycin during CaCl_2_‐induced differentiation. Scale bars, 50 μm. (D) Quantification of mean fluorescence intensity of autophagosomes. Data represent the mean of three independent experimental repeats (*n* = 3). All results are expressed as mean ± SD. *** *p* < 0.001 compared to the designated group. SD, standard deviation.
**Figure S5:** Dependence of PDK1 inhibition‐mediated amelioration of epidermal differentiation on autophagic flux. (A) Western blotting of differentiation markers (K10, INV, and LOR) and autophagy‐related proteins (Beclin‐1, LC3B, and p62) in NHEKs from young (34 year) and old (64 year) donors. Old NHEKs were treated with PDK1 inhibitors (100 nM of BX795, 100 nM of GSK2334470 [GSK], or 5 μg/mL of KT1) in the absence or presence of the Bafilomycin A1 (Bafilomycin, 100 nM) (B) Quantification of K10, INV, LOR, Beclin1, LC3B, and p62 levels. All results are expressed as mean ± SD. * *p* < 0.05; ** *p* < 0.01; *** *p* < 0.001 compared to the vehicle‐treated old (64 year) NHEKs. SD, standard deviation.
**Figure S6:** The HPLC chromatogram of CF. CF is predominately composed of KT (approximately 67% of total CF).
**Figure S7:** The effects of CF and its active compound, KT1, on differentiation, and autophagy in old donor‐derived NHEKs. CF (2 and 10 μg/mL) or KT1 (1, 2.5, and 5 μg/mL) treated to NHEKs from old donor for 48 h (A) Western blotting of differentiation, AKT/mTOR signalling, and autophagy markers in NHEKs. (B) Quantification of K10, INV, LOR, AKT phosphorylation, 4EBP1 phosphorylation, Beclin1, and LC3B levels. (C) Autophagosomes were evaluated by immunofluorescence images stained with autophagosomes of NHEK cells derived from old donors treated with CF. Scale bars, 50 μm. (D) The mean fluorescence staining intensity of autophagosomes. Data represent the mean of three independent experimental repeats (*n* = 3). All results are expressed as mean ± SD. * *p* < 0.05; ** *p* < 0.01; *** *p* < 0.001 compared to the vehicle‐treated NHEKs from 58 year donor. SD, standard deviation.
**Figure S8:** CF treatment rescues age‐related changes of DEJ and differentiation proteins in SE derived from old donors. (A) SE model sections were stained with H&E. The indicated proteins were detected by immunohistochemistry or immunofluorescence staining. Scale bars, 100 μm. (B) Quantification of epidermal thickness, Ki‐67‐positive cells, and the levels of COL17, Integrin β1, K10, FLG, INV, and LC3B using ImageJ software. Three representative images were analysed per skin equivalent, and image‐level values were averaged to yield one value per tissue for statistical analysis (*n* = 3 independent skin equivalents per group). All results are expressed as mean ± SD. * *p* < 0.05; ** *p* < 0.01; *** *p* < 0.001 compared to the vehicle‐treated old donors. SD, standard deviation.
**Figure S9:** Study flow chart. A randomized, vehicle‐controlled, double‐blind, intra‐individual study was conducted to compare the effects of 0.1% CF cream and vehicle control. Twenty‐four participants were enrolled, of whom four withdrew during follow‐up. Data from 20 participants were included in the final analysis.
**Table S1:** Formulations of CF cream.
**Table S2:** Two‐way repeated‐measures ANOVA of hydration, transepidermal water loss (TEWL), and transparency. *F*‐values and *p*‐values for the treatment × time interaction.

## Data Availability

All data are available in the main text or the Supporting Information, and further inquiries can be directed to the corresponding authors.
