# Supplementary material for: Inhibition of 3‐Phosphoinositide‐Dependent Kinase 1 Improves Age‐Associated Epidermal Dysfunction
Source: Exp Dermatol. 2026 Oct 30;35(11):e70379. doi: 10.1111/exd.70379 (PMC13722536; doi:10.1111/exd.70379)
Supplement: Supplementary file 1 [file EXD-35-e70379-s001.zip › exd70379-sup-0002-FigureS1-S9-TableS1-S2.docx]

**Supplementary Material**

**Inhibition of 3-Phosphoinositide-Dependent Kinase 1 Improves Age-Associated Epidermal Dysfunction**

Si-Young Cho^1†^, Hyung-Su Kim^1†^, Seung-Hun Kim^1^, Kyung-Mi Joo^1^, Sung Yeon Cho^1^, Sang Wook Son^2^, Heung-Soo Baek^1^, Hyoung-June Kim^1^, Jaeyoung Ko^1*^, A Young Kim^1*^

^1^Research and Innovation Center, AMOREPACIFIC, Yongin-si, Gyeonggi-do 17074, Korea

^2^Department of Dermatology, Korea University Ansan Hospital, Ansan 15355, Korea

Figure S1−S9

Table S2

**Supplementary Information legends**

**Figure S1.** Age-related changes of DEJ components and their effects on epidermal homeostasis in 3D SE derived from young and old donors. (A) SE sections were stained with H&E. The indicated proteins were detected by immunohistochemistry or immunofluorescence staining. Results are from one representative experiment out of three. Scale bars, 100 μm. (B) Quantification of epidermal thickness, Ki-67-positive cells, and the levels of COL17, Integrin β1, K10, FLG, INV, and LC3B using ImageJ software. Three representative images were analyzed per skin equivalent, and image-level values were averaged to yield one value per tissue for statistical analysis (n = 3 independent skin equivalents per group). All results are expressed as mean ± SD. ∗*P* < 0.05; ∗∗*P* < 0.01; ∗∗∗*P* < 0.001 compared to the young donors. SD, standard deviation.

**Figure S2**. Age-associated changes in keratinocyte differentiation during CaCl₂-induced differentiation of NHEKs. (A) Immunoblotting of differentiation markers in NHEKs isolated from young and aged donors after CaCl_2_ stimulation. The β-actin panel is identical to that shown in Figure S3A, as the proteins presented in both figures were analyzed using the same experimental samples and shared loading control. (B) Quantification of K10 and INV levels. Data are presented as a donor-level scatter plot, where each point represents an individual donor (n = 4 per age group). All results are expressed as mean ± SD. ** *P* <0.001 compared to the young donors. SD, standard deviation.

**Figure S3**. Age-associated changes in autophagy during CaCl₂-induced differentiation of NHEKs. (A) Immunoblotting of autophagy markers in NHEKs isolated from young and aged donors after CaCl_2_ stimulation. The β-actin panel is identical to that shown in Figure S2A, as the proteins presented in both figures were analyzed using the same experimental samples and shared loading control. (B) Quantification of ULK1 phosphorylation, Beclin1, and LC3B levels. Data are presented as a donor-level scatter plot, where each point represents an individual donor (n = 4 per age group). (C) Autophagosome formation visualized by immunofluorescence staining of LC3-positive structures in NHEK from young versus aged donors. Scale bars, 50 µm. (D) Quantitative fluorescence intensity of the autophagosome. Data represent the mean of three independent experimental repeats (n = 3). All results are expressed as mean ± SD. ** *P* <0.01; *** *P* <0.001 compared to the young donors. SD, standard deviation.

**Figure S4**. Autophagy inhibition impairs, whereas autophagy induction restores, keratinocyte differentiation during CaCl₂-induced differentiation conditions. (A) Western blotting of differentiation and autophagy markers in NHEKs. Bafilomycin A1 (Bafilomycin, 100 nM) was co-treated to inhibit autophagy in NHEKs from young donor. Rapamycin (250 nM) was used to induce autophagy in NHEKs from old donors. (B) Quantification of K10, INV, Beclin1, and LC3B levels. (C) Autophagosome formation visualized by immunofluorescence staining in NHEK from young and aged donors treated with bafilomycin or rapamycin during CaCl₂-induced differentiation. Scale bars, 50 μm. (D) Quantification of mean fluorescence intensity of autophagosomes. Data represent the mean of three independent experimental repeats (n = 3). All results are expressed as mean ± SD. *** *P* <0.001 compared to the designated group. SD, standard deviation.

**Figure S5.** Dependence of PDK1 inhibition-mediated amelioration of epidermal differentiation on autophagic flux. (A) Western blotting of differentiation markers (K10, INV, and LOR) and autophagy-related proteins (Beclin-1, LC3B, and p62) in NHEKs from young (34Y) and old (64Y) donors. Old NHEKs were treated with PDK1 inhibitors (100 nM of BX795, 100 nM of GSK2334470 [GSK], or 5 μg/ml of KT1) in the absence or presence of the Bafilomycin A1 (Bafilomycin, 100 nM) (B) Quantification of K10, INV, LOR, Beclin1, LC3B, and p62 levels. All results are expressed as mean ± SD. * P <0.05; ** P <0.01; *** P <0.001 compared to the vehicle-treated old (64Y) NHEKs. SD, standard deviation.

**Figure S6**. The HPLC chromatogram of CF. CF is predominately composed of KT (approximately 67 % of total CF).

**Figure S7**. The effects of CF and its active compound, KT1, on differentiation, and autophagy in old donor-derived NHEKs. CF (2 and 10 μg/ml) or KT1 (1, 2.5, and 5 μg/ml) treated to NHEKs from old donor for 48 hr (A) Western blotting of differentiation, AKT/mTOR signaling, and autophagy markers in NHEKs. (B) Quantification of K10, INV, LOR, AKT phosphorylation, 4EBP1 phosphorylation, Beclin1, and LC3B levels. (C) Autophagosomes were evaluated by immunofluorescence images stained with autophagosomes of NHEK cells derived from old donors treated with CF. Scale bars, 50 μm. (D) The mean fluorescence staining intensity of autophagosomes. Data represent the mean of three independent experimental repeats (n = 3). All results are expressed as mean ± SD. * *P* <0.05; ** *P* <0.01; *** *P* <0.001 compared to the vehicle-treated NHEKs from 58Y donor. SD, standard deviation.

**Figure S8.** CF treatment rescues age-related changes of DEJ and differentiation proteins in SE derived from old donors. (A) SE model sections were stained with H&E. The indicated proteins were detected by immunohistochemistry or immunofluorescence staining. Scale bars, 100 μm. (B) Quantification of epidermal thickness, Ki-67-positive cells, and the levels of COL17, Integrin β1, K10, FLG, INV, and LC3B using ImageJ software. Three representative images were analyzed per skin equivalent, and image-level values were averaged to yield one value per tissue for statistical analysis (n = 3 independent skin equivalents per group). All results are expressed as mean ± SD. * *P* <0.05; ** *P* <0.01; *** *P* <0.001 compared to the vehicle-treated old donors. SD, standard deviation.

**Figure S9**. Study flow chart. A randomized, vehicle-controlled, double-blind, intra-individual study was conducted to compare the effects of 0.1% CF cream and vehicle control. Twenty-four participants were enrolled, of whom four withdrew during follow-up. Data from 20 participants were included in the final analysis.

**Supplementary Table**

**Table S1**. Formulations of CF cream

| **Component** | **CF (% w/w)** | **Vehicle (% w/w)** |
| --- | --- | --- |
| Water | 92.375 | 92.475 |
| Dimethicone | 4 | 4 |
| Glyceryl stearate citrate | 1 | 1 |
| Dipotassium Glycyrrhizate | 1 | 1 |
| Cetearyl alcohol | 0.5 | 0.5 |
| polyaceylate-13 & polyisobutene | 0.5 | 0.5 |
| Citric acid monohydrate | 0.475 | 0.475 |
| Ethylhexylglycerin | 0.05 | 0.05 |
| CF | 0.1 | 0 |

**Table S2**. Two-way repeated-measures ANOVA of hydration, transepidermal water loss (TEWL), and transparency. F-values and p-values for the treatment × time interaction.

|  | **DF (DFn, DFd)** | **F-Value** | **P-Value** |
| --- | --- | --- | --- |
| Hydration | 2, 76 | 48.42 | <0.0001 |
| TEWL | 2, 76 | 12.88 | <0.0001 |
| Transparency | 2, 76 | 6.7 | 0.0021 |
